# Effect of the Samples’ Surface With Complex Microscopic Geometry on 3 × 3 Mueller Matrix Measurement of Tissue Bulks

**DOI:** 10.3389/fbioe.2022.841298

**Published:** 2022-03-09

**Authors:** Yi-Rong Liu, Wei-Zheng Sun, Jian Wu

**Affiliations:** ^1^ School of Medicine, Tsinghua University, Beijing, China; ^2^ Tsinghua Shenzhen International Graduate School, Tsinghua University, Shenzhen, China; ^3^ Tsinghua-Berkeley Shenzhen Institute, Tsinghua University, Shenzhen, China

**Keywords:** polarization imaging, surface with microscopic geometry, 3 × 3 Mueller matrix, physical models, clinical tissue bulks

## Abstract

The clinical *in vivo* tissue bulks’ surface is always coarse and shows a complex microscopic geometry which may affect the visual effect of polarization images and calculation of polarization parameters of the sample. To confirm whether this effect would cause identification difficulties and misjudgments on the target recognition when performed the polarization imaging based on 3 × 3 Mueller matrix measurement, cylindrical type and slope type physical models were used to study and analyze the effect of the surface with complex microscopic geometry on the polarization images. Then, clinical tumor bulk samples were used to interact with different sizes of patterns to simulate the different complex microscopic geometry and test the coarse surface effect on polarization images. Meanwhile, assessment parameters were defined to evaluate and confirm the variation between two polarization images quantitatively. The results showed that the polarization imaging of the sample surface with the complex microscopic geometry led to acceptable visual effect and limited quantitative variation on the value of polarization parameters and assessment parameters, and it caused no identification difficulties on target recognition, indicating that it is feasible to apply the polarization imaging based on 3 × 3 Mueller matrix measurement on clinical *in vivo* tissues with the complex microscopic geometry sample surface.

## Introduction

Polarization imaging technique is a promising noninvasive method to provide rich microstructural information about different types of the sample (KU et al., 2019; Ramella-Roman et al., 2020), primarily used as biomedical imaging applications (Xiao et al., 2019). It has been combined with microscopy (He et al., 2019) and endoscopy to help achieve precision and noninvasive medical detection and treatment, and polarization parameters have been proposed to explain distinctive pathological structures (Lu and Chipman, 1996; He et al., 2019) and help to detect human skin, esophageal, colorectal, and oral cancers and cervical carcinoma (Sheng et al., 2019) among others. Among the available polarization techniques, Mueller matrix imaging can provide comprehensive descriptions on the optical properties of biological samples (Pezzaniti and Chipman, 1995; Chung et al., 2002). The quantitative characterization of the Mueller matrix and polarization parameters (Ossikovski, 2009) provides a promising way to extract possible indicators for characteristic pathological microstructural features.

Nevertheless, we adopted the 3 × 3 Mueller matrix measurement in this study. Without the circular polarizations in the construction of 3 × 3 Mueller matrix measurement, it significantly simplifies the experimental geometry, which is particularly appropriate for *in vivo* polarimetry in the clinics (Forward et al., 2017; Khaliq et al., 2021). In addition, the experimental setup we adopted in this study requires less preparation of detected samples, it can measure the clinical tissue bulks with the coarse surface directly, and there is no need to make the sample sliced and flat (He et al., 2015; Wang et al., 2015; Dong et al., 2017). Since distinguishing the lesion region from normal tissue is always the priority, the 3 × 3 Mueller matrix and the backscattering configuration were used in this study to obtain the polarization images and parameters, which indicated less but enough polarization information for polarization measurement of clinical *in vivo* tissue bulks. However, the clinical tissues’ surface is coarse and shows a complex microscopic geometry which affects the propagation of polarized light in the sample, changing scattering and absorption events, and may affect the induced polarization parameters. In this article, we studied the feasibility of the 3 × 3 Mueller matrix measurement using clinical *in vivo* tissues with the complex microscopic geometry sample surface and demonstrated the quantitative variation of the effect on polarization images.

## Materials and Methods

### Theory

The polarization imaging technique can provide rich optical and microstructural information carried in the form of a Mueller matrix (Pezzaniti and Chipman, 1995; Chung et al., 2002; He et al., 2017) of detected samples. Since the relationship between microstructures of the sample and the specific Mueller matrix elements is unclear, Mueller matrix decomposition and transformation methods have been proposed to characterize specific properties of the samples (Azzam, 1978; Lu and Chipman, 1996; Ossikovski et al., 2008; Ossikovski, 2009; Ortega-Quijano and Arce-Diego, 2011; Ossikovski, 2011; Du et al., 2014; Chue-Sang et al., 2017; Liu et al., 2019; Ahmad et al., 2020). In this study, the polarization images and parameters from Mueller matrix polar decomposition (MMPD) (Lu and Chipman, 1996)and Mueller matrix transformation (MMT) methods (Xiao et al., 2019) were used to characterize the features of the sample:

$$M=M_{\Delta }M_{R}M_{D},$$ (1)

where ***M*** referred to the Mueller matrix of the sample, and ***M***_***Δ***_, ***M***_***R***_, and ***M***_***D***_ referred to depolarization, retardance, and diattenuation matrices, respectively. Then, the depolarization power (***Δ***), diattenuation power (***D***), and the value of linear retardance (***δ***) are calculated as:

 Δ=1—[(|a|+|b|+|c|)/3]; (2)

$$D=\left[1/m_{11}\right]\sqrt{m_{12}^{2}+m_{13}^{2}};$$ (3)

$$R=\cos^{-1}(tr\left(M_{R}\right)/2—1);$$ (4)

$$\delta =\cos^{-1}\left({\left\{{\left[m_{R21}+m_{R12}\right]}^{2}+{\left[m_{R11}+m_{R22}\right]}^{2}\right\}}^{1/2}-1\right),$$ (5)

where ***a***, ***b***, and ***c*** represented the eigenvalues of ***M***_***Δ***_, ***m***_***11***_ ***, m***_***12***_, and ***m***_***13***_ referred to elements of the ***M***, while ***m***_***R21***_, ***m***_***R12***_, ***m***_***R11***_, and ***m***_***R22***_ referred to elements of the ***M***_***R***_, and tr (***M***_***R***_) represented the trace of ***M***_***R.***_ Meanwhile, the parameter **A**, parameter **b** and parameter **t** from the MMT method are shown in (Eqs. 6–9), respectively.

$$A=2bt/\left(b^{2}+t^{2}\right);$$ (6)

$$b=\left(m_{22}+m_{33}\right)/2;$$ (7)

$$t=\sqrt{{\left(m_{22}—m_{33}\right)}^{2}+{\left(m_{23}+m_{32}\right)}^{2}}/2,$$ (8)

where parameter ***A*** characterized the aligned anisotropic microstructures, and parameter ***b*** indicated the depolarization properties of the samples (Xiao et al., 2019). These polarization parameters have been proved meaningful for differentiating certain biomedical features (Lu and Chipman, 1996), especially can provide optical and microstructural information concerning the differences between cancerous and healthy tissues (He et al., 2019).

### Methods

#### Materials

**FIGURE 1:**
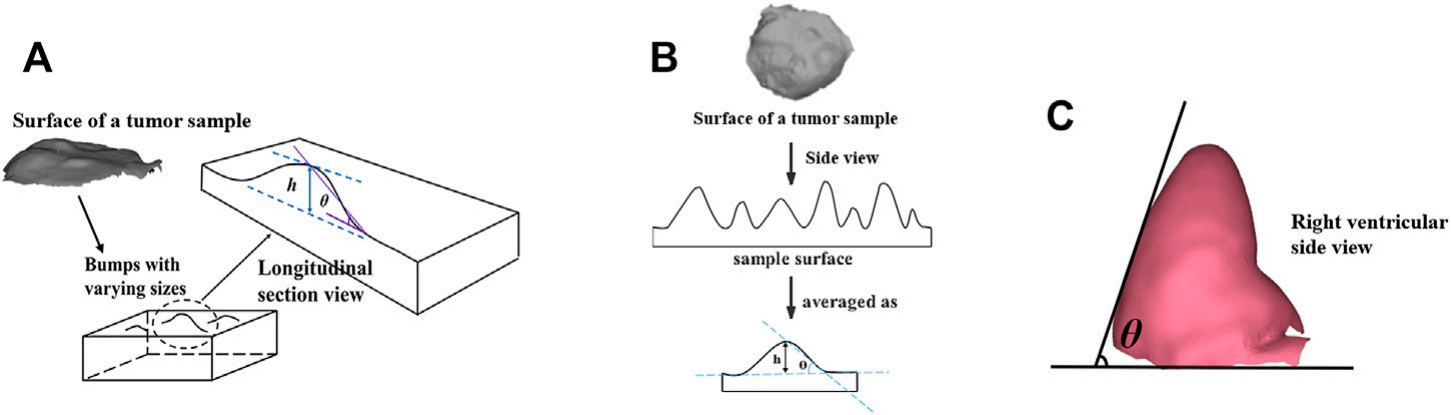
**(A)** Physical models of the samples or *in vivo* samples composed of lumps with varying sizes. **(B)** Schematic of the sample surface with complex microscopic geometry consisting of lumps with varying sizes, which were simplified with ***h/θ.* (C)** Positive view of the chicken heart physical model showing the ***θ*** measurement.

In total, two types of models were used in our study as follows:

Type 1: *Cylindrical type*: 1) Synclastic silk-spanned three-dimensional (3D)-printed cylinders with varying ***h***. 2) Chicken hearts were cut into cylinders with varying ***h***. Each sample measurement repeated three times to calculate the average value. All the measurements were used to simulate different lump’s ***h***, varying from 1 to 10 mm in 1 mm increments, which involved 10 samples.
Type 2: *Slope Type*: 1) Synclastic silk-spanned 3D-printed slope with varying slope angles ***θ***. 2) Chicken hearts were cut into a series of slopes with 5 mm fixed ***h***. The slopes on the right ventricular side of each sample were used (as Figure 1C shown). All the measurements were used to simulate different slope angles ***θ***, varying from 10° to 80° in 10° increments, which involved eight samples.

Then, nine tumor samples from The Second Hospital of Shenzhen were used to simulate the *in vivo* tissues’ status and shapes and verify the effect of *in vivo* tissues with the complex microscopic geometry sample surface on the polarized light imaging and parameters.

**FIGURE 2:**
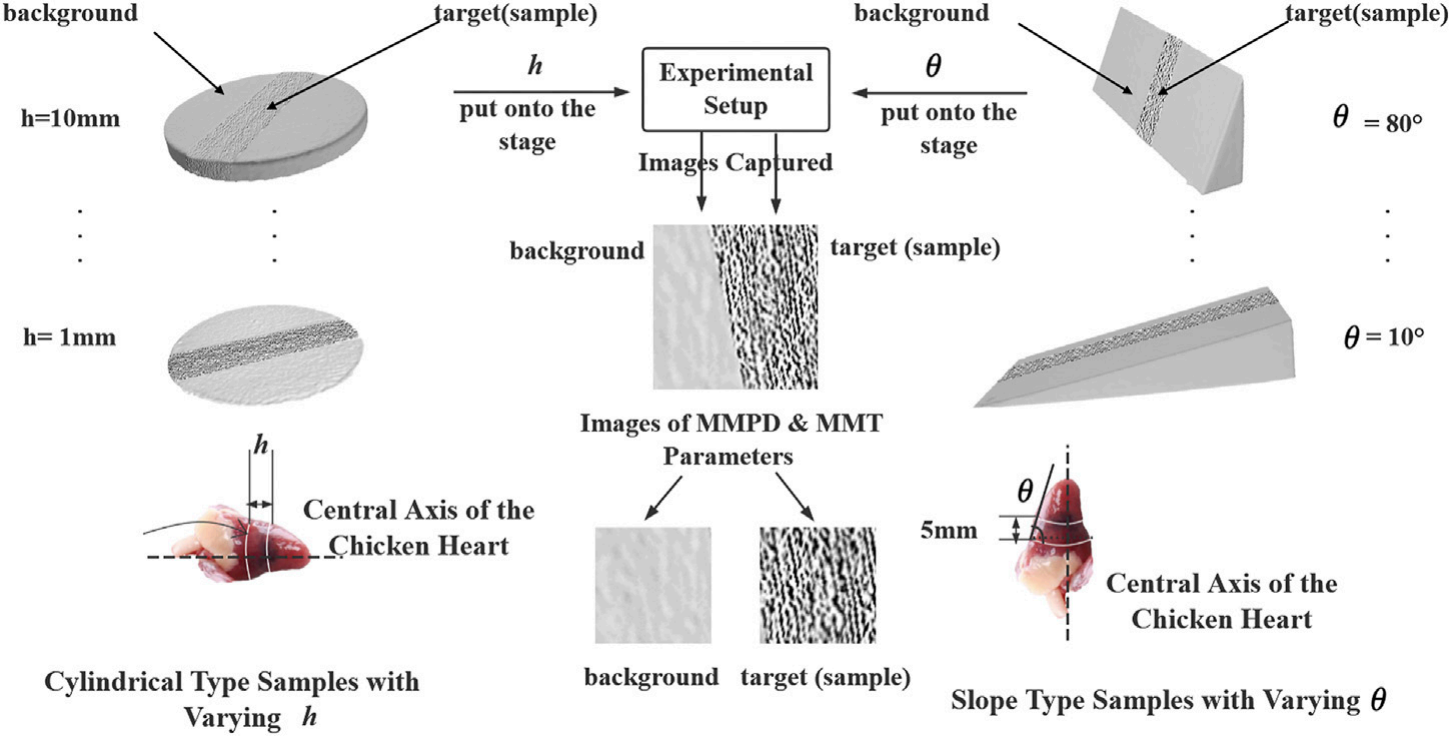
Schematic of the experiments in the research with physical models.

The schematic of experiments is shown in Figure 2, and the left and the right branch of the map described the experiments with the ***h*** and ***θ***, which involved both the Type 1 and Type 2 physical models.

#### Image Processing and Data Analysis

The 3 × 3 Mueller matrix (all the elements of the Mueller matrix were normalized by m11) of each sample was obtained first to calculate the polarization parameters, which included parameter ***Δ***, parameter ***D*** *,* and parameter ***δ*** calculated from the MMPD method and parameter ***A*** *,* parameter ***b*** *,* and parameter ***t*** calculated from the MMT method. For convenience and conciseness, they were all in a set of polarization parameters: Set ***V***:

V={Δ,D, δ,A,b,t}. (9)

We have calculated and tested all parameters listed in set ***V*** of each sample to compare and analyze which parameter presented the most significant differences during the samples’ ***h/θ*** changed, and polarization parameters were used to do the calculation separately.

For the first part of the experiments, Type 1 and Type 2 physical models with different ***h*** and ***θ*** were used. Since the *in vivo* tissues’ surface always presents a complex microscopic geometry, consisting of many lumps with varying sizes (shown as Figure 1A), and the larger image contains the more complex microscopic geometric details, which were simplified as different height (represented with ***h*** in a subscript form) and steepness (represented as ***θ*** in a subscript form) factors to affect the polarization imaging and parameters calculation (as shown in Figure 1B). So, ***h*** and ***θ*** were used in the physical models to evaluate the effect of *in vivo* tissues with the complex microscopic geometry sample surface on the polarized light imaging and parameters.

The average values of Set ***V*** of background/target with different ***h*** were expressed as ***V***_***(h)bn***_ and ***V***_***(h)tn***_ (***n*** represented the serial number of the samples); the average values of Set ***V*** of background/target with different ***θ*** were expressed as ***V***_***(θ) bn***_ */* ***V***_***(θ)tn***_ (***n*** represented the serial number).

**FIGURE 3:**
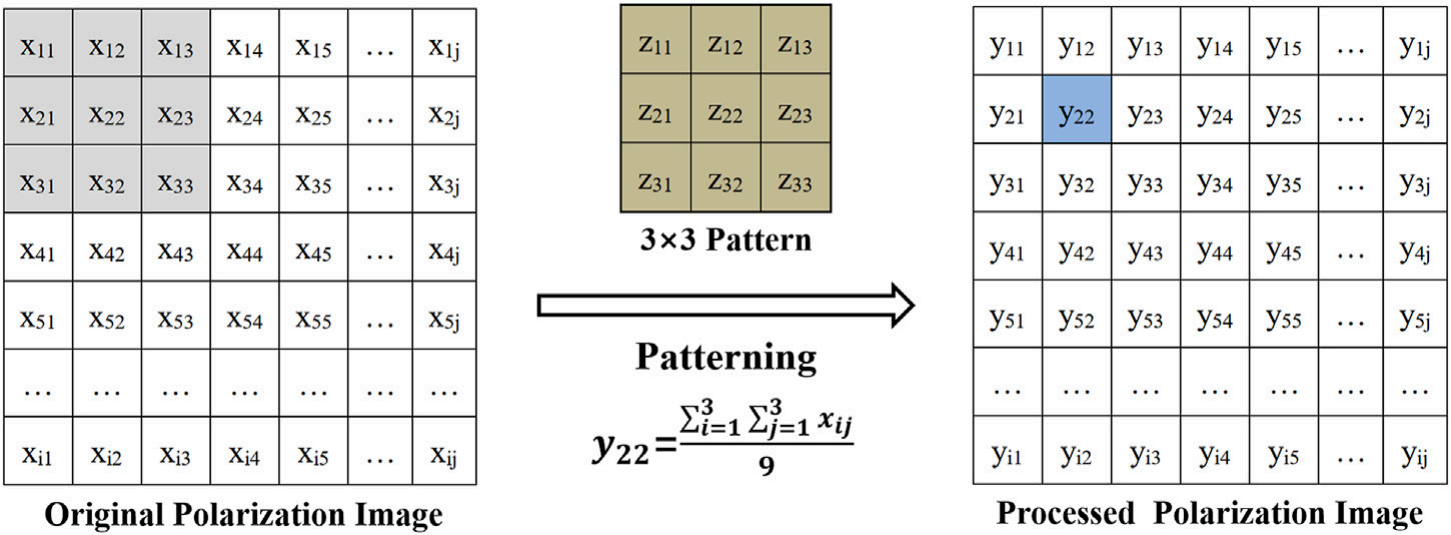
Schematic of the experiments in the research with tumor samples (e.g., 3 × 3 patterning process) and the determination of ***h/θ*** of the sample surface with complex microscopic geometry.

For the second part of the experiments, we used clinical tumor bulk samples whose surface presents a complex microscopic geometry to study the effect on polarization images and parameters. In order to simulate this effect, different sizes of patterns (***z*** × ***z, z*** = 1,3,5,9,15) were applied to the polarization images of the samples. The larger the patterns we used, the larger the images were affected. In addition, as mentioned previously, the larger the images we detect, the more different the microscopic geometry details it contains, that is, the more ***h/θ*** factors to affect the polarization imaging and parameters calculation (shown as Figure 3), so we used different sizes of patterns to control the different combinations of ***h*** and ***θ*** contained in the images involved in polarization parameters calculation:

$$y_{ij}=\left(\sum_{i=1}^{z}\overset{z}{\underset{j=1}{\sum }}x_{ij}\right)/z^{2},$$ (10)

in which ***i*** and ***j*** were indexes of the matrices. The pattern matrix ***Z*** was used to interact with the original image (matrix ***X,*** the matrix without interacting with the patterns), obtaining the processed images (matrix ***Y,*** which represented the matrix after interacting with the patterns). Then, the average values of Set ***V*** of background/target of processed images after interacting with the patterns were expressed as ***V***_***ba***_ and ***V***_***ta***_ (***a*** = 1,3,5,9,15, represented the size of the pattern matrix ***Z***).

Then, four ways were used to evaluate and confirm the differences, which include 1) the polarization images (in a nonquantitative or visual effect way), 2) assessment parameter ***∆V*** in Eqs. 13, 14 (in a quantitative way), 3) assessment parameter ***∆C*** in Eq. 16 (in a quantitative way), and 4) assessment parameter ***P*** in Eq. 17 (in a quantitative way)

$$V_{tn}=1/n\sum_{k=1}^{n}V_{tk\prime };$$ (11)

$$V_{bn}=1/n\overset{n}{\underset{k=1}{\sum }}V_{bk\prime };$$ (12)

$$\Delta V_{b}=V_{b\max}—V_{b\min};$$ (13)

$$\Delta V_{t}=V_{t\max}—V_{t\min};$$ (14)

$$C=\left|V_{b}—V_{t}\right|;$$ (15)

$$\Delta C=C_{\max}—C_{\min};$$ (16)

$$P=\Delta C/\left(C_{\max}+C_{\min}\right),$$ (17)

in which the subscript ***b*** refers to the background*,* ***t*** refers to the target, and *max* refers to the maximum value among the samples, while *min* refers to the minimum value among the samples*.* Suppose that *p* < 0.5 is a threshold for a good display that ***C*** was an acceptable change, and it could be considered that the effect from the sample surface with different microscopic geometry would cause little identification difficulties on target or tumor recognition.

### 3 × 3 Mueller Matrix Experimental System

**FIGURE 4:**
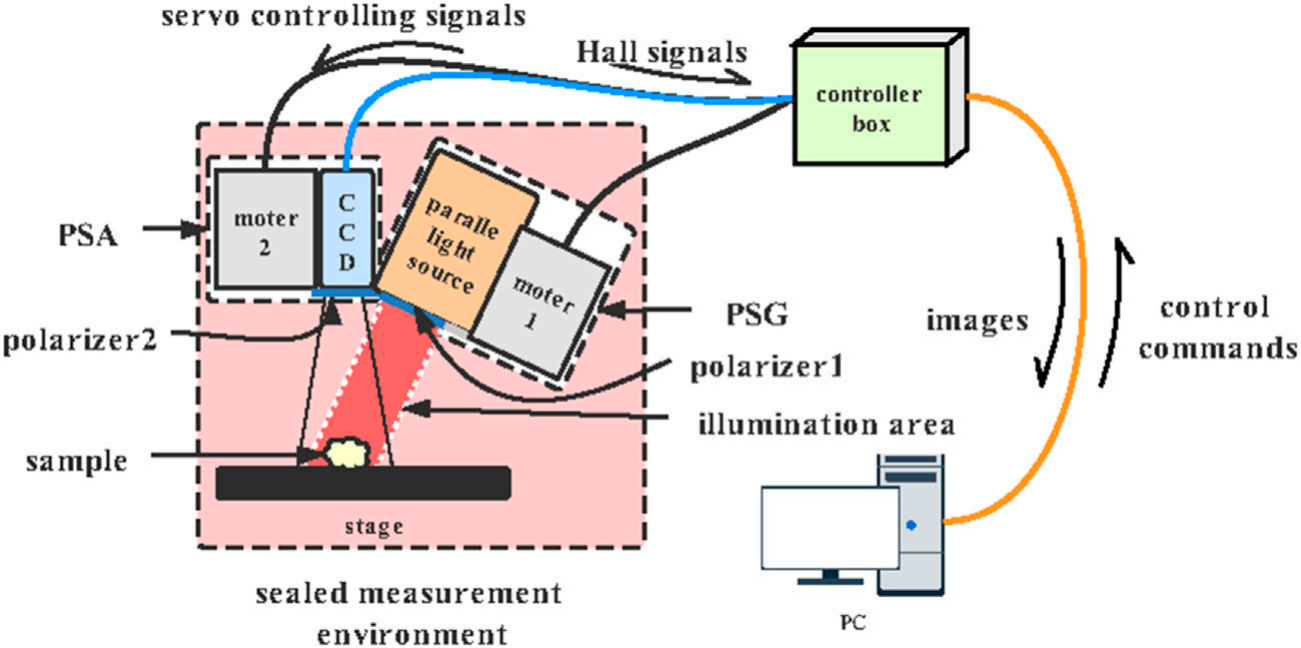
Schematic diagram of the experimental setup.

The 3 × 3 Mueller matrix polarization experimental system in a backscattering mode is shown in Figure 4, which consists of a sealed measurement environment, a controller box, and a personal computer (PC). The polarization state generator (PSG), polarization state analyzer (PSA), and the sample stage are in the sealed measurement environment to ensure that the capturing process is performed in a dark environment. The incident light from a parallel light source (630 nm, Tele Optics, China) passes through the polarizer (P1), providing a circular illumination area of 50 mm diameter. The photons backscattered from the sample pass through the analyzing polarizer (P2) and then recorded using a monochrome industry camera (DMK33UX265, The Imaging Source, Germany) to acquire the polarization images at 60 frames per second. A total of two DC servo motors (DSEM series, Motec, China) rotated the polarizers to generate different PSG states and PSA states. Also, there is a 20° angle between the incident light and the CCD camera axis to avoid the sample’s surface reflection.

## Results

### Effect of *h* and *θ* on Polarization Images and Parameters’ Value of Cylindrical/Slope Physical Models

According to the methods presented previously, Type 1 and Type 2 physical models were used to study the effect of ***h*** and ***θ*** on polarization images and parameters’ value. Each sample’s polarization parameters were calculated from MMT and MMPD methods, and assessment parameters ***∆V***_***b***_ ***, ∆V***_***t,***_ ***∆C*** *,* and ***P*** were calculated.

**FIGURE 5:**
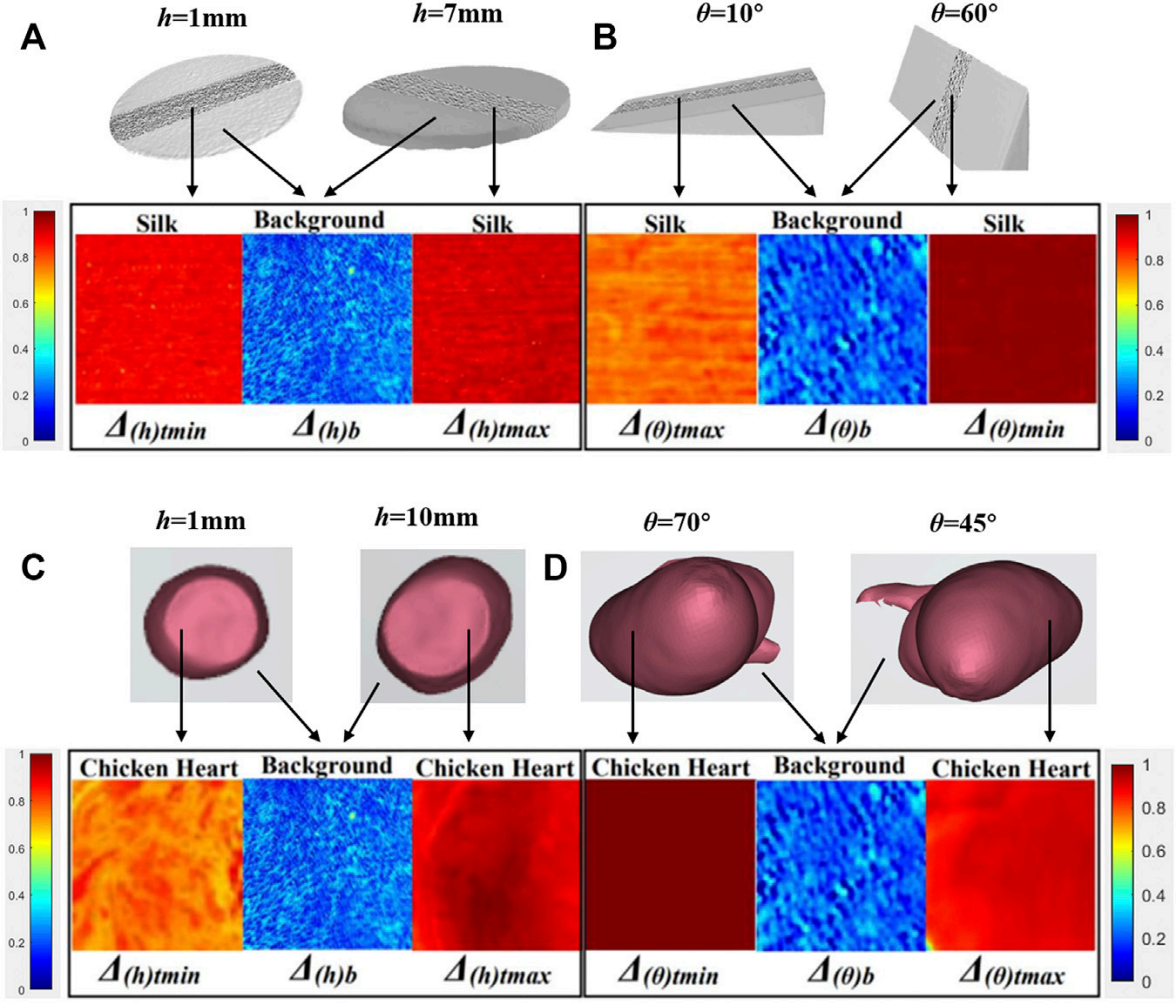
Parameter ***Δ*** images affected by ***h*** and ***θ*** from Type 1 and Type 2 physical models. **(A)** Type 1 physical models—silk samples with ***h*** varying. **(B)** Type 2 physical models—silk samples with ***θ*** varying. **(C)** Type 1 physical models—chicken heart samples with ***h*** varying. **(D)** Type 2 physical models—chicken heart samples with ***θ*** varying.

The results are shown in Table 1 and Figure 5.

**TABLE 1:** ***∆Δ*** (h/***θ***), ***∆C***, and ***P*** of parameter Δ of physical models affected by ***h*** and ***θ***.

Parameter		Study of *h* (Type 1 physical models)		Study of *θ* (Type 2 physical models)	
		*Silk*	*Chicken heart*	*Silk*	*Chicken heart*
*Δ*	*ΔΔ*_*t*_	0.04	0.24	0.20	0.25
	***ΔC***	0.03	0.10	0.21	0.25
	***P***	0.02	0.22	0.16	0.19

Figure 5 showed the maximum variation of parameter ***Δ*** images with the Type 1 and Type 2 physical models’ ***h***/***θ*** varying. Each image was composed of three sub-images. The intermediate sub-image referred to the background region, while the side sub-images referred to the regions of physical models. From sub-images on both sides, the difference between images of ***Δ***_***(h/θ)t max***_ and ***Δ***_***(h/θ)t min***_ could be observed, which yielded **Δ*Δ***_***(h/θ)t***_ according to Eqs. 2, 14 of silk samples, and the contrast between the background and regions of physical models could be seen and yielded ***ΔC***_***(h/θ)***_ according to Eq. 16. Meanwhile, from Table 1, the values of ***P***_***(h/θ)***_ of the Type 1 and Type 2 physical models with ***h***/***θ*** varying calculated from Eq. 17 were also listed. Although a different visual effect could be observed, it caused no confusion between the target and background recognition.

### Effect of the Tumor Samples’ Surface With Different Microscopic Geometry on Polarization Images and Parameters

A total of nine tumor samples from the clinics were used to study the effect of the samples’ surface with different microscopic geometry on polarization images and parameters with the matrix ***Z*** interacted with the original images. As a result, less significant or little difference in ***ΔC*** after the patterning processes was observed, making the images remain almost the same as the original images, which indicated that the samples’ surface with different microscopic geometry had little effect on 3 × 3 Mueller matrix measurement.

Specifically, the parameter ***Δ*** showed the maximum variation during the patterning processes. Different pattern modes were performed on the polarization images to yield a series of average ***h*** and ***θ,*** as shown in Figure 3, and some differences during the patterning processes were noted.

The results were shown as follows: Table 2 and Table 3 showed the variations of the ***∆Δ***, ***∆C***, and ***P*** of the parameter ***Δ*** and parameter ***b*** *,* which displayed the maximum variations among the polarization parameters (***∆Δ*** in Table 2, and ***∆b*** in Table 3) of the tumor samples with different pattern modes.

**TABLE 2:** ***∆Δ***, ***∆C***, and ***P*** of parameter ***Δ*** of the tumor samples’ surface with complex microscopic geometry.

Parameter		Lymphoma group	Liver cancer group	Breast cancer group
*Δ*	Δ*Δ*_*t*_	0.03	0.05	0.02
	***ΔC***	0.04	0.05	0.02
	***P***	0.03	0.08	0.02

**TABLE 3:** ***∆b***, ***∆C***, and ***P*** of parameter ***b*** of the tumor samples’ surface with complex microscopic geometry.

Parameter		Lymphoma group	Liver cancer group	Breast cancer group
*b*	Δ*b*_*t*_	0.02	0.03	0.02
	***ΔC***	0.07	0.07	0.05
	***P***	0.05	0.12	0.05

Parameter ***Δ***, which showed the maximum variations between the original and the processed images with different pattern modes in all tumor samples, indicated that the development of tumors was strongly related to the change of cellular density of the samples. Therefore, Table 2 showed the results of the tumor samples’ surface with microscopic geometry represented by different ***h*** and ***θ*** on parameter ***Δ*** where the variations were maximum with different pattern modes. **Δ*Δ***_***t***_ refers to the maximum variation of the tumor samples between the original and the processed parameter ***Δ*** images calculated from Eqs. 2, 14. At the same time, ***∆C*** shows the maximum contrast between the original and the processed parameter ***Δ*** images’ target and background of the tumor sample, calculated from Eq. 16. ***P*** demonstrated the difference between original and processed parameter ***Δ*** images calculated from Eq. 17, and *p* ≤ 0.22, which indicated that it would cause no misjudgment of the target or tumor recognition.

Parameter ***b***, another parameter characterizing the depolarization process, showed significant variations between the original and the processed images with different pattern modes in all tumor samples, and the results are listed in Table 3. Furthermore, **Δ*b***_***t***_ refers to the maximum variation of the tumor samples between the original and the processed parameter ***b*** images calculated from Eqs. 7, 14. As shown in Table 3, *p* ≤ 0.12, which also indicated that it would cause no misjudgment of the target or tumor recognition.

**FIGURE 6:**
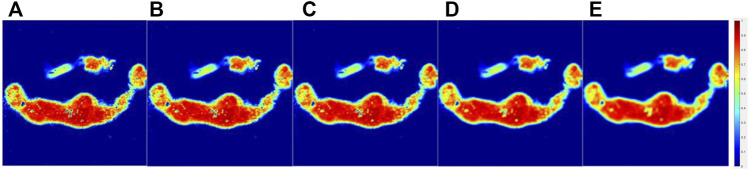
Original parameter ***Δ*** images of the lymphoma group **(A)** and processed images interacted with 3 × 3**(B)**, 5 × 5**(C)**, 9 × 9**(D)**, and 15 × 15**(E)** masks.

Figure 6 showed the parameter ***Δ*** images of the original and processed images interacted with 3 × 3, 5 × 5, 9 × 9, and 15 × 15 patterns of the lymphoma tumor group samples’ surface with microscopic geometry represented by different ***h*** and ***θ***; some blur was observed, and detailed information lost in Figure 6E compared with the original image in Figure 6A, and it could be easily seen that after interacted with different sizes of patterns, the processed images became more blurred with the size of the pattern matrix ***Z*** grown. Nevertheless, it could be easily differentiated between the tumor and background.

**FIGURE 7:**
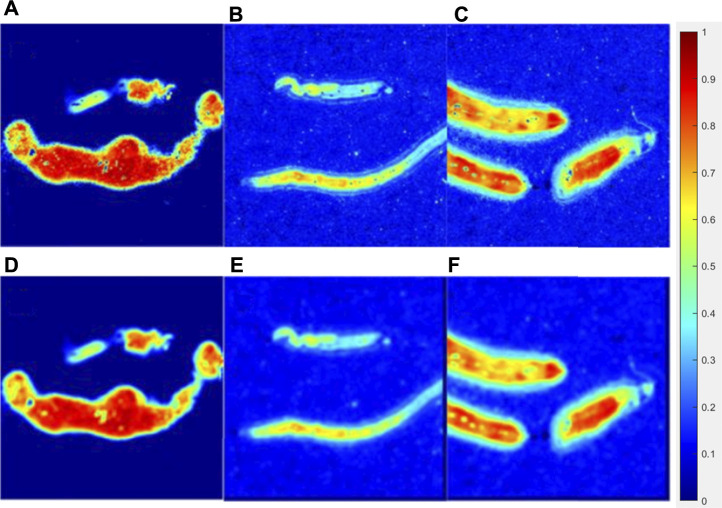
Original parameter ***Δ*** image of the lymphoma group **(A)**, liver cancer group **(B)**, breast cancer group **(C),** and processed images interacted with a 15 × 15 **(E)** pattern of each group **(D,E,F)**.

**FIGURE 8:**
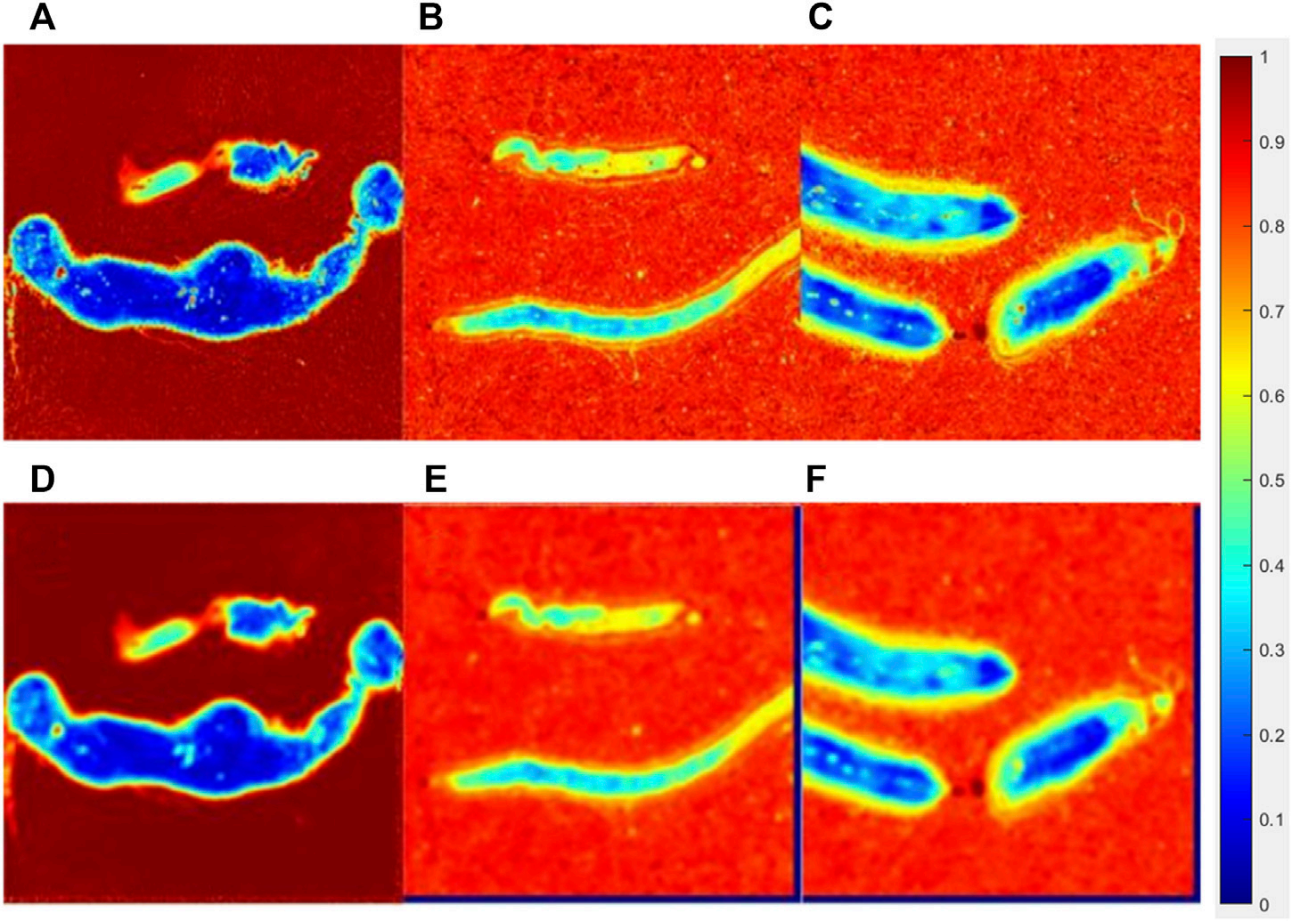
Original parameter ***b*** image of the lymphoma group **(A)**, liver cancer group**(B)**, breast cancer group**(C),** and processed images interacted with a 15 × 15**(E)** pattern of each group **(D,E,F)**.

Figure 7 showed the original parameter ***Δ*** images (the upper line) and processed images interacted with a 15 × 15 pattern (the bottom line) of the lymphoma group, liver cancer group, and breast cancer group, while Figure 8 showed the original images of parameter ***b*** (the upper line) and processed images interacted with a 15 × 15 pattern (the bottom line) of three groups. The patterning process made the images blur. The ***∆C*** and ***P*** of the processed images have a limited variation compared with those of the original images, which could be quantitatively measured in Tables 2, 3. However, it made no difference for tumor recognition.

In conclusion, in the experiments on assessing the effect of ***h*** and ***θ*** on polarization images and values of parameters of physical models, it was found that ***∆C*** ≤ 0.29 with a mean ***P*** of 0.15, while ***∆C*** ≤ 0.07 with the mean ***P*** of 0.06 in the experiments on the effect of the detected tumor samples’ surface with different microscopic geometry. The samples’ surface with the complex microscopic geometry made a negligible blur effect on distinguishing tumors or targets but caused little qualitative change on polarization images and identification difficulties on target recognition, and it is feasible to apply the polarization imaging based on 3 × 3 Mueller matrix measurement on *in vivo* tissues with the complex microscopic geometry sample surface.

## Discussion

It is proposed that ***∆C*** and ***P*** were feasible indicators to show the variation bringing up from the effect of the detected samples’ surface with different microscopic geometry. The ***∆C*** values from the experiments on the effect of ***h*** and ***θ*** on polarization images and values of parameters of physical models were greater than those from the experiments on the tumor samples’ surface with different microscopic geometry; the reason might be that the variables ***h*** and ***θ*** from Type 1 and Type 2 physical models were much larger than those of the detected tumor sample specimens’ surface with complex microscopic geometry (e.g., the ***h*** of tumor samples <2 mm, while Type 1 and Type 2 physical models’ ***h*** <10 mm). However, in the second part of the study, since the larger the patterns, the larger the interacted image windows which contain more combinations of ***h***/***θ*** to affect the polarization images and parameters, different sizes of patterns were used on clinical tumor tissue bulks to simulate different combinations of ***h***/***θ***, which is a qualitative control to give a rough illustration, which still need further research to determine the quantitative relation between the size of the patterns and coarseness of the surface.

Nevertheless, since the maximum variations in ***∆C*** and ***P*** from the experiments on the effect of the detected tumor samples’ surface with complex microscopic geometry introduced no confusion and misjudgment when it came to differentiate the tumors or targets from the background, it was feasible to use the clinical or *in vivo* tissues with complex microscopic geometry performing the polarization imaging detection, and it made no difference to identify the tumors or targets, which paved the way to provide an opportunity to develop polarization imaging detection on clinical samples.

The effect of the detected samples’ surface with complex microscopic geometry on polarization parameters’ values and images was studied in this article, and the results validated that this effect led to little qualitative change on polarization images and identification difficulties on target recognition, which laid the foundation for the powerful and noninvasive polarization imaging technique applied in clinical samples.

## Data Availability

The raw data supporting the conclusion of this article will be made available by the authors, without undue reservation.

## References

[B1] Ahmad I. Khaliq A. Iqbal M. Khan S. (2020). Mueller Matrix Polarimetry for Characterization of Skin Tissue Samples: a Review. Photodiagnosis Photodynamic Ther. 30, 101708. 10.1016/j.pdpdt.2020.101708 32145374

[B2] Azzam R. M. A. (1978). Propagation of Partially Polarized Light through Anisotropic media with or without Depolarization: A Differential 4 × 4 Matrix Calculus. J. Opt. Soc. Am. 68 (12), 1756–1767. 10.1364/josa.68.001756

[B3] Chue-Sang J. Bai Y. Stoff S. Gonzalez M. Holness N. A. Gomes J. (2017). Use of Mueller Matrix Polarimetry and Optical Coherence Tomography in the Characterization of Cervical Collagen Anisotropy. J. Biomed. Opt. 22 (8), 086010. 10.1117/1.jbo.22.8.086010 PMC5997002 28853246

[B4] Chung J. R. Baba J. S. DeLaughter A. H. Cote G. L. (2002). “Development and Use of a Novel Automated Mueller Matrix Polarization Imaging System for *In-Vivo* Imaging of Lesions,” in Optical Biopsy IV 4613, 111–117. SPIE. 10.1117/12.465235

[B5] Dong Y. Qi J. He H. He C. Liu S. Wu J. (2017). Quantitatively Characterizing the Microstructural Features of Breast Ductal Carcinoma Tissues in Different Progression Stages by Mueller Matrix Microscope. Biomed. Opt. Express 8 (8), 3643–3655. 10.1364/boe.8.003643 28856041 PMC5560831

[B6] Du E. He H. Zeng N. Sun M. Guo Y. Wu J. (2014). Mueller Matrix Polarimetry for Differentiating Characteristic Features of Cancerous Tissues. J. Biomed. Opt. 19 (7), 076013. 10.1117/1.jbo.19.7.076013 25027001

[B7] Forward S. Gribble A. Alali S. Lindenmaier A. A. Vitkin I. A. (2017). Flexible Polarimetric Probe for 3 × 3 Mueller Matrix Measurements of Biological Tissue. Sci. Rep. 7 (1), 11958–12012. 10.1038/s41598-017-12099-8 28931853 PMC5607295

[B8] He C. Chang J. Hu Q. Wang J. Antonello J. He H. (2019). Complex Vectorial Optics through Gradient index Lens Cascades. Nat. Commun. 10 (1), 4264–4268. 10.1038/s41467-019-12286-3 31537802 PMC6753074

[B9] He C. He H. Chang J. Dong Y. Liu S. Zeng N. (2015). Characterizing Microstructures of Cancerous Tissues Using Multispectral Transformed Mueller Matrix Polarization Parameters. Biomed. Opt. Express 6 (8), 2934–2945. 10.1364/boe.6.002934 26309757 PMC4541521

[B10] He H. He C. Chang J. Lv D. Wu J. Duan C. (2017). Monitoring Microstructural Variations of Fresh Skeletal Muscle Tissues by Mueller Matrix Imaging. J. Biophotonics 10 (5), 664–673. 10.1002/jbio.201600008 27160958

[B11] Khaliq A. Ashraf S. Gul B. Ahmad I. (2021). Comparative Study of 3 X 3 Mueller Matrix Transformation and Polar Decomposition. Opt. Commun. 485, 126756. 10.1016/j.optcom.2021.126756

[B12] K. U. S. Mahato K. K. Mazumder N. (2019). Polarization-resolved Stokes-Mueller Imaging: a Review of Technology and Applications. Lasers Med. Sci. 34 (7), 1283–1293. 10.1007/s10103-019-02752-1 30830559

[B13] Liu T. Lu M. Chen B. Zhong Q. Li J. He H. (2019). Distinguishing Structural Features between Crohn's Disease and Gastrointestinal Luminal Tuberculosis Using Mueller Matrix Derived Parameters. J. Biophotonics 12 (12), e201900151. 10.1002/jbio.201900151 31465142

[B14] Lu S.-Y. Chipman R. A. (1996). Interpretation of Mueller Matrices Based on Polar Decomposition. J. Opt. Soc. Am. A. 13 (5), 1106–1113. 10.1364/josaa.13.001106

[B15] Ortega-Quijano N. Arce-Diego J. L. (2011). Depolarizing Differential Mueller Matrices. Opt. Lett. 36 (13), 2429–2431. 10.1364/ol.36.002429 21725434

[B16] Ossikovski R. (2009). Analysis of Depolarizing Mueller Matrices through a Symmetric Decomposition. J. Opt. Soc. Am. A. 26 (5), 1109–1118. 10.1364/josaa.26.001109 19412227

[B17] Ossikovski R. Anastasiadou M. Ben Hatit S. Garcia-Caurel E. De Martino A. (2008). Depolarizing Mueller Matrices: How to Decompose Them? Phys. Stat. Sol. (A) 205 (4), 720–727. 10.1002/pssa.200777793

[B18] Ossikovski R. (2011). Differential Matrix Formalism for Depolarizing Anisotropic media. Opt. Lett. 36 (12), 2330–2332. 10.1364/ol.36.002330 21686010

[B19] Pezzaniti J. L. Chipman R. A. J. O. E. (1995). Mueller Matrix Imaging Polarimetry. Opt. Eng. 34 (6), 1558–1568. 10.1117/12.206161

[B20] Ramella-Roman J. C. Saytashev I. Piccini M. (2020). A Review of Polarization-Based Imaging Technologies for Clinical and Preclinical Applications. J. Opt. 22 (12), 123001. 10.1088/2040-8986/abbf8a

[B21] Sheng W. Li W. Qi J. Liu T. He H. Dong Y. (2019). Quantitative Analysis of 4× 4 Mueller Matrix Transformation Parameters for Biomedical Imaging. Photonics 6, 34. Multidisciplinary Digital Publishing Institute. 10.3390/photonics6010034

[B22] Wang Y. He H. Chang J. Zeng N. Liu S. Li M. (2015). Differentiating Characteristic Microstructural Features of Cancerous Tissues Using Mueller Matrix Microscope. Micron 79, 8–15. 10.1016/j.micron.2015.07.014 26280279

[B23] Xiao Y. Rivaz H. Chabanas M. Fortin M. Machado I. Ou Y. (2019). Evaluation of MRI to Ultrasound Registration Methods for Brain Shift Correction: the CuRIOUS2018 challenge. IEEE Trans. Med. Imaging 39 (3), 777–786. 10.1109/TMI.2019.2935060 31425023 PMC7611407

